# The Added Value of a High CT Coronary Artery Calcium Score in the Management of Patients Presenting with Acute Chest Pain vs. Stable Chest Pain

**DOI:** 10.3390/jcdd9110390

**Published:** 2022-11-13

**Authors:** Rafael Hitter, Amir Orlev, Itshak Amsalem, Nir Levi, Talya Wolak, Rivka Farkash, Naama Bogot, Michael Glikson, Arik Wolak

**Affiliations:** 1 Jesselson Integrated Heart Center, Shaare Zedek Medical Center, Faculty of Medicine, Hebrew University of Jerusalem, Jerusalem 9112102, Israel; 2 Department of Internal Medicine, Shaare Zedek Medical Center, Faculty of Medicine, Hebrew University of Jerusalem, Jerusalem 9112102, Israel; 3 Department of Radiology, Shaare Zedek Medical Center, Faculty of Medicine, Hebrew University of Jerusalem, Jerusalem 9112102, Israel

**Keywords:** coronary CT angiography, calcium score, chest pain

## Abstract

Background: Contrast computerized tomography (CT) scan is occasionally aborted due to a high coronary artery calcium score (CACS). For the same CACS in our clinical practice, we observed a higher occurrence of severe coronary artery disease (CAD) in patients with acute chest pain (ACP) compared to patients with stable chest pain (SCP). Since it is known that ACP differs in many ways from SCP, the aim of this study was to compare the predictive value of a high CACS for the diagnosis of severe CAD between ACP and SCP patients. Methods: This single center observational retrospective study included consecutive patients who underwent cardiac CT for chest pain and were found to have a CACS of >200 Agatston units. Patients were divided into two groups, ACP and SCP. Severe CAD was defined as ≥70% stenosis on coronary CT angiography or invasive coronary angiography. Baseline characteristics and final diagnosis of severe CAD were compared. Results: The cohort included 220 patients, 106 with ACP and 114 with SCP. ACP patients had higher severe CAD rates (60.4% vs. 36.8%; *p* < 0.001). On multivariate analysis including cardiac risk factors, CACS > 400 au (OR = 2.34 95% CI [1.32–4.15]; *p* = 0.004) and ACP (OR = 2.54 95% CI [1.45–4.45]; *p* = 0.001) were independent predictors of severe CAD. The addition of the clinical setting of ACP added significant incremental predictive value for severe stenosis. Conclusion: A high CACS is more associated with severe CAD in patients presenting with ACP than SCP. The findings suggest that the CACS could impact the management of patients during the scan.

## 1. Introduction

Triage of patients presenting with chest pain remains challenging. The initial approach is comprised of a clinical assessment that includes history, physical examination, and electrocardiography (ECG), as well as cardiac biomarkers [1,2,3]. Recently, coronary computed tomography angiography (CCTA) has been emerging as a suitable approach to further improve risk stratification for cardiovascular events, especially for patients presenting with atypical chest pain along with negative biomarkers and negative findings on ECG [1,2,3,4,5].

Although not mandatory, a typical cardiac computed tomography (CT) protocol includes a non-contrast-enhanced scan for coronary artery calcium (CAC) scoring prior to the CCTA scan. Yet, in the setup of symptomatic patients, CAC scoring by itself is hardly used for diagnosis. Instead, in practice, the use of coronary artery calcium score (CACS) for patients presenting with chest pain has evolved as an adjunct to CCTA and serves several purposes. First, it is used as an anatomical reference that helps to limit the scan length and as a result to reduce radiation exposure [6]. In addition, since a high CAC burden reduces the diagnostic performance of the scan, it can be used to predict potentially non-interpretable studies [7]. Lastly, assessment of CAC can be used as a marker for higher risk of significant coronary artery disease (CAD) [8]. Therefore, in real life setups, a finding of high CACS used to deter the following step of contrast injection and in these cases; the patients were usually referred for further investigation, straight to invasive coronary angiography (ICA), or other means of investigation such as functional testing. Due to recent significant technological improvements in both scanner hardware and software, the challenge of dealing with high coronary calcium burden has lessened and the CACS threshold for aborting the contrast injection has been raised [9]. However, it is still reasonable to avoid contrast injection in patients with a high probability of significant stenosis.

Patients with chest pain can be divided into two groups based on the chronicity of their complaints: acute chest pain (ACP) and stable chest pain (SCP). These chest pain syndromes differ in many ways. They have different etiologies and features, different risk stratifications and prognoses, and are managed and treated differently [1,2,3,10]. Accurately assessing these patients is vital since patients with chest pain, especially patients with acute coronary syndrome (ACS), have high morbidity and mortality rates [1,2,3]. Since we noted in our clinical practice that by the end of the investigation the same CAC burden was associated with higher rates of significant CAD among ACP patients in comparison to SCP patients, we sought to further study this phenomenon. Therefore, the aim of this work was to investigate the value of a high CACS to predict significant stenosis in ACP vs. SCP patients.

## 2. Materials and Methods

Study design and population: In this observational retrospective study, we examined the medical records of consecutive patients who underwent a cardiac CT study at the Shaare Zedek Medical Center between the 1 January 2015 and the 31 May 2019. The study was approved by the Shaare Zedek Medical Center Institutional Review Board; written informed consent was waived. Patients were referred to a cardiac CT from the Emergency department (ED), other in-house departments (predominantly the cardiology department), and from outpatient clinics. Patients who met the following criteria were included: 1. Cardiac CT study was done for the investigation of chest pain, or angina equivalent symptoms; 2. CACS was >200 Agatston Units (au); 3. CAC scoring was followed by CCTA or ICA which was conducted for further investigation. Exclusion criteria included: 1. Cardiac CT study done for an indication other than the evaluation of chest pain or angina equivalent symptoms; 2. Positive diagnostic criteria for myocardial infarction at the time of the CT study; 3. ECG changes indicative of ischemia (e.g., ST depressions); 4. Prior percutaneous coronary intervention (PCI) or coronary artery bypass surgery (CABG). Since there are no explicit definitions of acute and stable chest pain in the current chest pain guidelines [3], acute chest pain patients were defined as patients presenting with chest pain in the ED, and stable chest pain patients were defined as patients who presented with chest pain in outpatients’ clinics. ACP patients were evaluated according to the guidelines of acute coronary syndromes [1] and SCP according to the guidelines of chronic coronary syndromes [2].

Data collection: Baseline characteristics and medication history were collected from the patients’ electronic medical records. The categorical cardiac risk factors examined were age, gender, smoking habits, family history of CAD, diabetes mellitus, hyperlipidemia, hypertension, previous cerebral vascular accident (CVA) and peripheral vascular disease (PVD). The medications that were examined included aspirin, P2Y12 inhibitors, statins, beta blockers, and angiotensin-converting-enzyme inhibitors (ACE-I) or Angiotensin II receptor blockers (ARB). Only medications that were taken >1 month before the CT study were included.

CACS and CCTA protocol: All data was acquired on a dual-source CT scanner (SOMATOM Flash or SOMATOM Force, Siemens Healthineers, Erlangen, Germany). All acquisition and reconstruction protocols (spiral, sequential or high pitch spiral flash) adhered to the Society of Cardiovascular Computed Tomography guidelines for the performance and acquisition of coronary computed tomographic angiography [6]. Our CT laboratory clinical routine included an initial calcium score scan followed by CCTA.

Endpoints: The primary endpoint of this study was the presence of significant CAD defined as ≥50% stenosis for the left main coronary artery and ≥70% stenosis for any of the other coronary arteries on CCTA or ICA. Studies have shown a high concordance between CCTA and ICA [11,12,13], therefore, both modalities were used in this study. Stenosis was visually analyzed and quantified. When both CCTA and ICA were performed, findings on ICA were used to measure the level of stenosis. The secondary endpoint was defined as revascularization by means of PCI or CABG.

Statistical analysis: Continuous variables were expressed as mean ± standard deviation and compared using the *t*-test, if distributed normally. Non-normally distributed continuous variables were expressed as median [interquartile range] and compared with the Mann–Whitney U test. Quantile-quantile plots were generated to determine normal distribution. Categorical variables were expressed as No. (percentages) of patients and were compared using the Pearson chi-square test. Multivariate analysis was used to identify independent predictors of severe CAD. We constructed a logistic regression model that included age, gender, clinical cardiac risk factors (smoking history, hypertension, family history of CAD, diabetes, hyperlipidemia, hypertension, previous CVA and PVD), a CACS of >400 au and setting (ACP vs. SCP). The incremental value of added data to a model was defined as a significant increase in the global chi square. Two-sided *p* value < 0.05 was considered statistically significant. Statistical analyses were performed using SPSS.

## 3. Results

**Figure 1:**
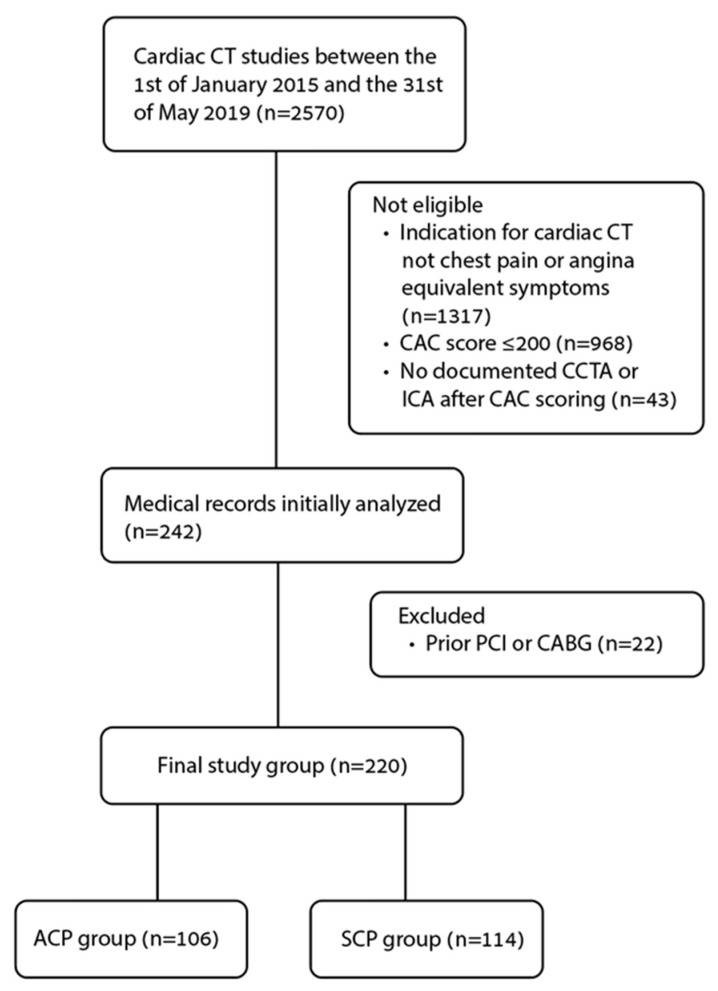
Flowchart of included/excluded patients, and study groups. ACP: acute chest pain, CABG: coronary artery bypass grafting, CAC: coronary artery calcium, CAD: coronary artery disease, PCI: percutaneous coronary intervention, SCP: stable chest pain.

There were 2570 patients who underwent cardiac CT study between the 1 January 2015 and the 31 May 2019. Of them, 1253 underwent the CT scans for the indication of chest pain or angina equivalent symptoms. In 968 of the cases, the CACS was ≤200 au and another 43 were found ineligible due to absent documented CCTA or ICA after CAC scoring. As a result, 242 patients were found eligible for initial analysis of the data (Figure 1). Based on the electronic medical records, 22 patients were excluded due to prior coronary intervention. The remaining 220 patients were divided into the two study groups, ACP and SCP (as defined above). 106 patients with ACP and 114 with SCP were included in the final statistical analysis.

Baseline characteristics, medication and imaging: Baseline clinical characteristics of the population are displayed in Table 1, stratified by the two study groups. The demographic characteristics and cardiac risk factors of the patients were similar except for a higher smoking rate in the ACP group (42.5% vs. 27.2%; *p* = 0.017). Data regarding medication history was available for 215 patients (97.7%). There were no significant differences between the groups. The median CAC score (450 vs. 408; *p* = 0.371), and the frequency of a CACS >400 au (58.5% vs. 51.8%; *p* = 0.316) and a CACS >1000 au (21.7% vs. 17.5%; *p* = 0.438) did not differ significantly either. ACP patients underwent significantly more ICA (68.9% vs. 42.1%; *p* < 0.001) and significantly less CTA (54.7% vs. 78.1%; *p* < 0.001) than SCP patients. All 106 patients (100%) presenting with ACP had new onset chest pain or angina equivalent symptoms starting <1 month before the cardiac CT study while all 114 patients (100%) presenting with SCP had symptoms lasting ≥1 month before the study. Of the patients presenting with SCP, 75% had symptoms lasting >2 months before the CT study.

**Figure 2:**
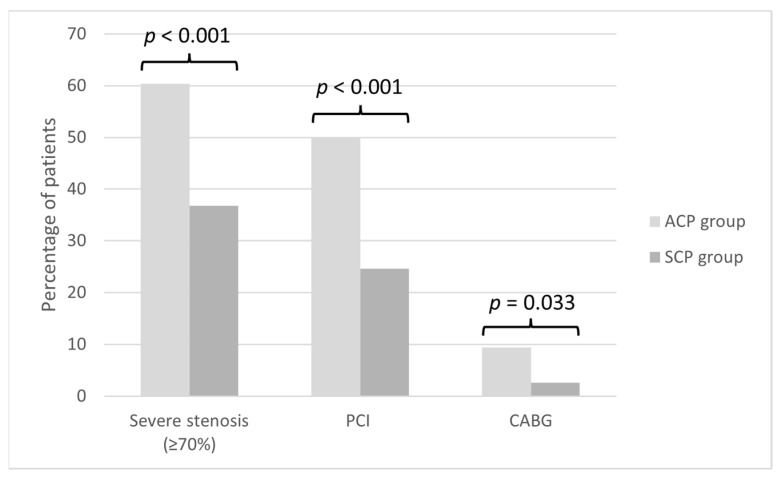
Rates of Severe stenosis, PCI and CABG stratified by the study groups. ACP: acute chest pain, CABG: coronary artery bypass grafting, PCI: percutaneous coronary intervention, SCP: stable chest pain.

Endpoints: Of the 220 patients included in the cohort, 106 (48.2%) were found to have ≥70% stenosis in at least one of the coronary arteries. Sixty-four (60.4%) ACP patients and 42 (39.6%) SCP patients had severe CAD (*p* < 0.001). Additionally, ACP patients underwent more PCI (50% vs. 24.6%; *p* < 0.001) and CABG (9.4% vs. 2.6%; *p* = 0.033) than the SCP group (Figure 2). Of the 106 patients with severe CAD, 64 (60%) had single vessel disease, 31 (29%) had double vessel disease and 11 (10%) had triple vessel disease.

Multivariate analysis: In a multivariate analysis adjusted by age, gender, any cardiac risk factors (as described above), CACS of >400 au and the clinical setting (ACP vs. SCP), the independent predictors of severe CAD were ACP (OR = 2.54 95% CI [1.45–4.45]; *p* = 0.001) and a CACS of >400 au (OR = 2.34 95% CI [1.32–4.15]; *p* = 0.004). To assess the incremental predictive value of CACS > 400 au and the clinical setting of ACP for severe stenosis, we constructed multiple logistic regression models (Table 2).

**Figure 3:**
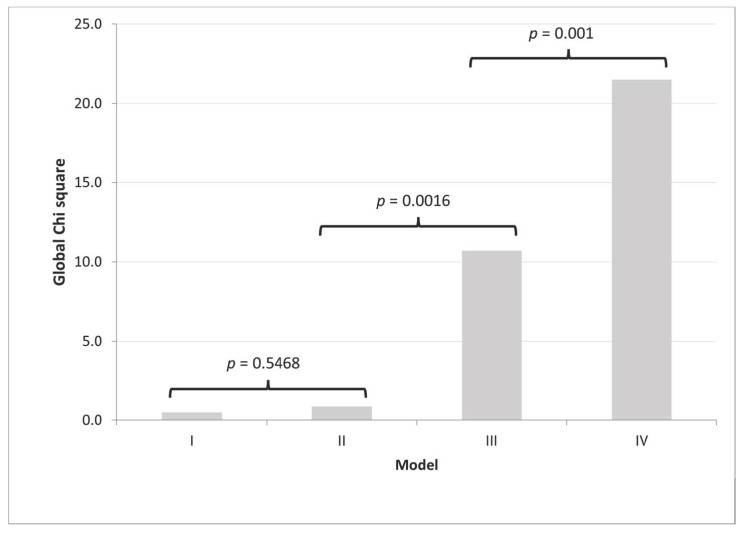
The incremental value of clinical setting (ACP) over clinical risk factors and CACS > 400 au resulted in significant increase in the global chi square. Among patients with a high CACS, the clinical setting adds significantly to the prediction of severe CAD. ACP: acute chest pain, au: Agatston Units, CACS: coronary artery calcium score, CAD: coronary artery disease. Model I: age + gender, Model II: age + gender + cardiac risk factors, Model III: age + gender + cardiac risk factors + CACS > 400 au, Model IV: age + gender + cardiac risk factors + CACS > 400 au + ACP. cardiac risk factors: indicate any of the established clinical cardiac risk factors including smoking history, hypertension, family history of coronary artery disease, diabetes, hyperlipidemia, hypertension, previous cerebral vascular accident and peripheral vascular disease.

As seen shown in Figure 3, there was no significant increase of the global chi square when adding any cardiac risk factors (Model II) to a model adjusted by age and gender (Model I). However, the addition of CACS > 400 au (Model III) to Model II, resulted in a significant increase of the global chi square (from 0.9 to 10.7; *p* = 0.0016), and subsequent addition of clinical setting (Model IV) to Model III resulted in further significant increase of the global chi square (from 10.7 to 21.5; *p* = 0.001).

## 4. Discussion

In this study, we found that in patients referred to a cardiac CT for the assessment of CAD with CACS of >200 au, the clinical setup of ACP was significantly associated with severe CAD compared to patients presenting with SCP. Interestingly, baseline characteristics did not differ significantly between the two groups except for higher smoking rates in the ACP group. ACP patients underwent more PCI and CABG compared to SCP patients. Additionally, in patients with a CACS of >200 au, the usual cardiac risk factors like age, gender, smoking history, hypertension, family history of CAD, diabetes, hyperlipidemia, hypertension, and previous CVA and PVD, were not found to be independently associated with higher rates of severe CAD, whereas ACP and a CACS of >400 au were. Although CACS > 400 au was independently associated with severe CAD, the clinical setup of ACP had a higher incremental value for predicting severe CAD.

Previous studies have investigated the use of CACS in the setting of ACP and SCP [14,15,16,17,18,19,20,21,22]. However, to our knowledge, none of these studies have compared CACS’ predictive value for severe CAD between both types of chest pain syndromes. Previous studies evaluating the diagnostic value of CACS in the setting of ACP in the ED in low- to intermediate-risk patients have shown high sensitivities (91–100%) and negative predictive values (NPV) (97–100%), but modest specificities (38–64%) and positive predictive values (PPV) (7.4–48%) [14,15,16,17,18,19]. Most studies demonstrated the added value of CACS in the assessment algorithm for ACP, mostly due to its high NPV [14,15,16,17,19]. They suggest that patients with a CACS of 0 can safely be discharged. The ROMICAT II Trial [18] negated this value and found that CACS offers no incremental diagnostic value for ACP patients, especially since CCTA is such a safe and efficient tool. For patients with a low likelihood of CAD presenting with SCP, CAC scoring was recommended by the 2010 NICE Guidance as the initial diagnostic test in the algorithm to assess their disease [20]. A CACS of zero was used to rule out significant CAD and a CACS of >400 au was used as a cutoff to deter CCTA and recommend alternative techniques such as coronary functional imaging or ICA. However, the updated 2016 NICE Guidance [21] recommends that all patients with SCP should first be investigated with CCTA regardless of the CACS due to case reports [22] of severe CAD with a CACS of zero.

Most previous studies did not find added value in the use of CACS to predict significant CAD due to its modest specificities and PPV [14,15,16,17,18,19]. However, Budoff et al. [23] incorporated CACS in a model for prediction of significant CAD in symptomatic patients. They found that CACS has incremental diagnostic value, in conjunction with pretest probability, in the prediction of significant CAD. Yet, this study did not differentiate between ACP and SCP. Winther et al. [24] developed a similar model for patients with chronic chest pain.

**Figure 4:**
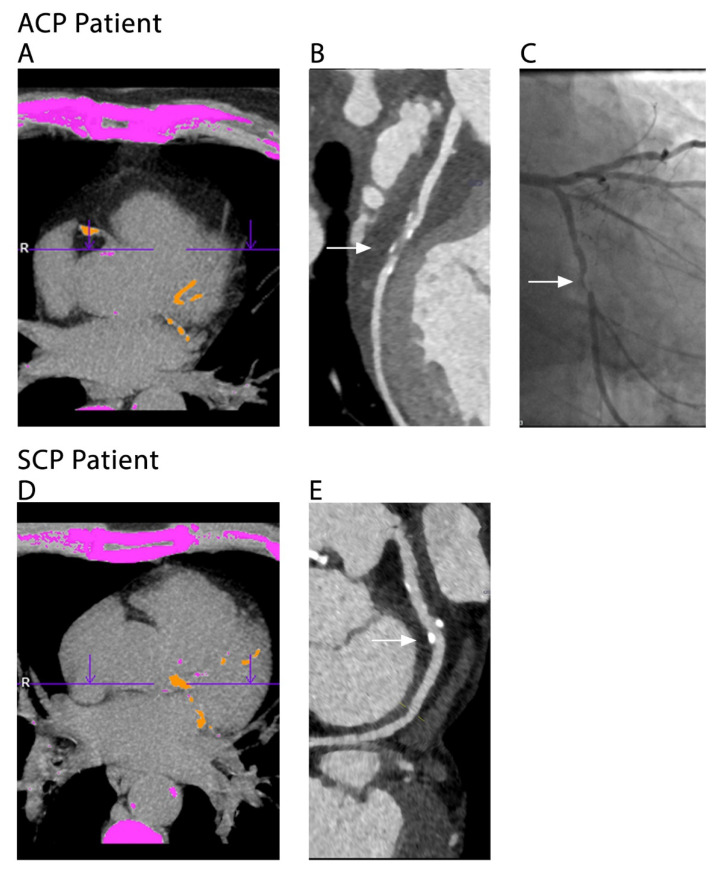
CT scan examples of an ACP and a SCP patient. (**A**–**C**) 67-year-old male ACP patient with a history of hypertension, hyperlipidemia and smoking. (**A**) CACS scan of 461 au. (**B**) CCTA shows a severe calcified lesion in the circumflex artery (CX) (arrow). (**C**) Subsequent ICA demonstrates the same severe lesion in the CX (arrow). (**D**,**E**) 65-year-old male SCP patient with a history of hyperlipidemia and smoking. (**D**) CACS scan of 418 au. (**E**) CCTA shows a non-obstructive calcified lesion in the CX. ICA was not performed since it was not indicated. ACP: acute chest pain, au: Agatston Units, CACS: coronary artery calcium score, CCTA: coronary CT angiography, CX: circumflex artery, ICA: invasive coronary angiography, SCP: stable chest pain.

**Figure 5:**
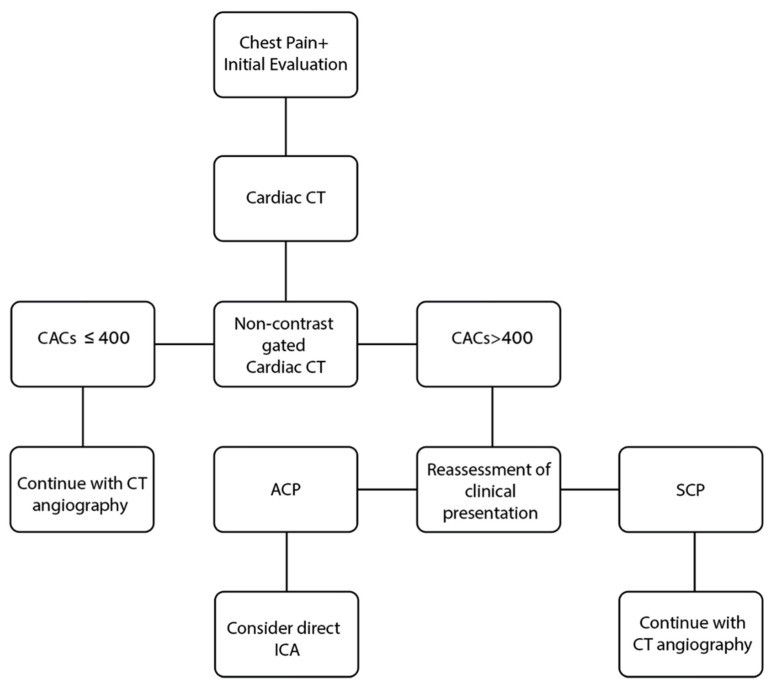
Proposed algorithm for the evaluation of chest pain along with negative cardiac biomarkers and negative findings on ECG. ACP: acute chest pain, CACS: coronary artery calcium score, ECG: electrocardiogram, ICA: invasive coronary angiography, SCP: stable chest pain.

The aim of our study was not to evaluate the use of CACS as a stand-alone diagnostic tool but to use this measurement in context of the clinical setup and to identify a group of patients with a higher pretest probability after the initial CACS scan and before continuing with CCTA, namely ACP patients. The results of this study indicate that in patients with a high CACS and similar baseline characteristics (i.e., medical and treatment history), the clinical setup of ACP was associated with severe CAD, whereas SCP was associated with severe CAD to a much lesser degree (Figure 4). Our findings add to the notion that clinical setup and patient population should impact the interpretation of imaging tools, thus improving its efficiency and yield [25]. Although some CT labs skip the initial non-contrast gated scan and CAC scoring, we suggest that the obtained information from the CAC scoring is important for the clinical management of the patient at that moment in time, while the patient is lying in the CT scanner. There should be a synergy between the clinical presentation of the patient and the imaging, and that synergy should guide cardiovascular imaging cardiologists not only at the time of interpretation of the scan but throughout the entire management of the patient. This could have real-life clinical implications in the assessment-algorithm of ACP patients. Based on the findings of this study that a CACS > 400 au and the clinical setup of ACP have an incremental value for predicting severe CAD, we propose the algorithm depicted in Figure 5. However, it should be noted that the algorithm is confined by the limitations of the study. Further stratification of this group could raise the predictive value of CACS for severe CAD even higher. It is also important to note that CT technology has improved since previous studies, and further study is necessary to determine its effectiveness in detecting CAC and thus its predictive diagnostic value.

Limitations: Some limitations of this study need to be addressed. First, this study is limited due to its retrospective nature and observational design at a single center. Furthermore, the study comprises of a small number of patients. Further study with a larger study sample is warranted to confirm the results. Another limitation is that ACP patients underwent significantly more ICA and significantly less CCTA compared to SCP patients. However, as described above, studies have shown a high concordance between CCTA and ICA [11,12,13]. This probably occurred due to clinical intuition and direct referral of ACP patients to ICA after a high CACS on the initial scan.

## 5. Conclusions

A high CACS is significantly more associated with severe CAD in patients presenting with ACP compared to patients presenting with SCP. ACP is also independently associated with severe CAD. The findings of this study suggest that a high CACS should impact the decision whether to proceed with CCTA when assessing ACP patients. They might benefit from being referred directly to ICA and avoid the excess radiation and technical difficulties that come along with CCTA.

## Figures and Tables

**Table 1:** Baseline characteristics, medication and imaging characteristics.

	ACP Group (n = 106)	SCP Group (n = 114)	*p* Value
**Age**	64.6 ± 9.6	66.4 ± 9.5	0.152
**Female**	33 (31.1%)	29 (25.4%)	0.348
**Family history of CAD**	25 (23.6%)	21 (18.4%)	0.347
**History of smoking**	45 (42.5%)	31 (27.2%)	0.017
**Hypertension**	69 (65.1%)	70 (61.4%)	0.571
**Hyperlipidemia**	75 (70.8%)	85 (74.6%)	0.526
**Diabetes mellitus**	32 (30.2%)	39 (34.2%)	0.524
**Previous CVA**	6 (5.7%)	8 (7.0%)	0.68
**PVD**	4 (3.8%)	5 (4.4%)	0.819
**Medication history**			
**Aspirin**	54 (50.9%)	50/109 (45.9%)	0.457
**P2Y12 Inhibitor**	8 (7.5%)	9/109 (8.3%)	0.847
**Statins**	65 (61.3%)	69/109 (63.3%)	0.764
**Beta blockers**	30 (28.3%)	32/109 (29.4%)	0.864
**ACEI or ARB**	58 (54.7%)	53/109 (48.6%)	0.371
**Imaging**			
**CAC score (au)**	450 [286–860]	408 [284–754]	0.371
**CAC score >400 au**	62 (58.5%)	59 (51.8%)	0.316
**CAC score >1000 au**	23 (21.7%)	20 (17.5%)	0.438
**CCTA**	58 (54.7%)	89 (78.1%)	<0.001
**ICA**	73 (68.9%)	48 (42.1%)	<0.001

Categorical variables are expressed as No. (percentages), continuous variables are expressed as mean ± standard deviation and non-normally distributed continuous variables are expressed as median [interquartile range]. Variables with missing data have a denominator shown as No./total No. (percentage). ACP: acute chest pain, ACEI: angiotensin-converting-enzyme inhibitors, ARB: angiotensin II receptor blockers, au: Agatston Units, CAC: coronary artery calcium; CAD: coronary artery disease, CCTA: coronary CT angiography, CVA: cerebral vascular accident, ICA: invasive coronary angiography, PVD: peripheral vascular disease, SCP: stable chest pain.

**Table 2:** Multivariate analysis for predictors of ≥70% coronary artery stenosis.

	Model I (Age + Gender)		Model II (Age + Gender + Any Cardiac Risk Factors)		Model III (Age + Gender + Any Cardiac Risk Factors + CACS > 400 au)		Model IV (Age + Gender + Any Cardiac Risk Factors + CACS > 400 au + ACP)	
	OR [CI]	*p* Value	OR [CI]	*p* Value	OR [CI]	*p*-Value	OR [CI]	*p* Value
**age**	0.99 [0.96–1.02]	0.498	0.99 [0.96–1.02]	0.535	0.99 [0.96–1.02]	0.359	0.99 [0.96–1.02]	0.599
**gender**	1.01 [0.55–1.88]	0.966	1.01 [0.54–1.86]	0.987	1.22 [0.64–2.33]	0.541	1.09 [0.56–2.13]	0.794
**any risk factor**			1.44 [0.44–4.70]	0.55	1.16 [0.34–3.89]	0.815	1.04 [0.30–3.59]	0.946
**CACS > 400 au**					2.42 [1.38–4.24]	0.002	2.34 [1.32–4.15]	0.004
**ACP**							2.54 [1.45–4.45]	0.001

ACP: acute chest pain, au: Agatston Units, CACS: coronary artery calcium score, CI: confidence interval, OR: odds ratio. cardiac risk factors: indicate any of the established clinical cardiac risk factors including smoking history, hypertension, family history of coronary artery disease, diabetes, hyperlipidemia, hypertension, previous cerebral vascular accident and peripheral vascular disease.

## Data Availability

Not applicable.
